# Supplementary material for: Nutritional Status Is Not a Predictor of Anaphylaxis Severity in a Pediatric Cohort: A Retrospective Analysis
Source: Nutrients. 2025 Sep 22;17(18):3023. doi: 10.3390/nu17183023 (PMC12472751; doi:10.3390/nu17183023)
Supplement: Supplementary file 1 [file nutrients-17-03023-s001.zip › Supplementary Table S3.pdf]

Supplementary Table S3. Post hoc powers for BMI subgroups

**Subgroup**	**Post hoc power**
Underweight	0.061
Normal weight	0.441
Overweight	0.092
Obese	0.074
Underweight + Normal	0.248
Overweight + Obese	0.195
