# Supplementary material for: Nutritional Status Is Not a Predictor of Anaphylaxis Severity in a Pediatric Cohort: A Retrospective Analysis
Source: Nutrients. 2025 Sep 22;17(18):3023. doi: 10.3390/nu17183023 (PMC12472751; doi:10.3390/nu17183023)
Supplement: Supplementary file 1 [file nutrients-17-03023-s001.zip › Supplementary Table S4.pdf]

Supplementary Table S4. Multivariate regression analyses for predictors of anaphylaxis severity

Predictor	Model 1 (Ordinal, BMI percentile)	Model 2 (Binary, BMI percentile)	Model 3 (Ordinal, BMI categories)	Model 4 (Binary, BMI categories)
BMI percentile	1.01 (0.998–1.02), p = 0.11	1.01 (1.00–1.02), p = 0.30	—	—
BMI category	—	—	1.58 (0.96–2.59), p = 0.07	1.48 (0.86–2.54), p = 0.15
Age	0.98 (0.92–1.04), p = 0.49	0.98 (0.92–1.05), p = 0.62	0.98 (0.92–1.04), p = 0.46	0.98 (0.92–1.05), p = 0.58
Sex	0.65 (0.36–1.19), p = 0.16	0.77 (0.41–1.46), p = 0.43	0.69 (0.38–1.26), p = 0.23	0.81 (0.43–1.52), p = 0.50
Asthma	0.76 (0.40–1.47), p = 0.41	0.86 (0.43–1.75), p = 0.68	0.73 (0.38–1.41), p = 0.35	0.83 (0.41–1.69), p = 0.60
Atopic dermatitis	0.55 (0.31–0.98), p = 0.04	0.57 (0.31–1.03), p = 0.06	0.54 (0.31–0.95), p = 0.03	0.55 (0.30–1.00), p = 0.05

Legend: Multivariate regression analyses examining predictors of anaphylaxis severity. Models 1–2 included BMI as a percentile; Models 3–4 included BMI as categorical nutritional status. Ordinal models used the full WAO scale (grades 1–5); binary models dichotomized severity into non-severe (1–3) vs. severe (4–5). All models were adjusted for age, sex, asthma, and atopic dermatitis. Odds ratios (OR) with 95% confidence intervals (CI) and p-values are reported.
