# Supplementary material for: Nutritional Status Is Not a Predictor of Anaphylaxis Severity in a Pediatric Cohort: A Retrospective Analysis
Source: Nutrients. 2025 Sep 22;17(18):3023. doi: 10.3390/nu17183023 (PMC12472751; doi:10.3390/nu17183023)
Supplement: Supplementary file 1 [file nutrients-17-03023-s001.zip › Supplementary Table S5.pdf]

Supplementary Table S5. Multivariate regression models characteristics

Model characteristic	Model 1 (Ordinal, BMI percentile)	Model 2 (Binary, BMI percentile)	Model 3 (Ordinal, BMI categories)	Model 4 (Binary, BMI categories)
Number of observations	199	199	199	199
Df residuals	190	193	190	193
Df model	5	5	5	5
Log-likelihood	-194.36	-128.06	-193.99	-127.52
LL-Null	-198.98	-130.79	-198.98	-130.79
LLR p-value	0.100	0.361	0.076	0.256
Pseudo-R^2	0.023	0.021	0.025	0.025
