# Supplementary material for: Nutritional Status Is Not a Predictor of Anaphylaxis Severity in a Pediatric Cohort: A Retrospective Analysis
Source: Nutrients. 2025 Sep 22;17(18):3023. doi: 10.3390/nu17183023 (PMC12472751; doi:10.3390/nu17183023)
Supplement: Supplementary file 1 [file nutrients-17-03023-s001.zip › Supplementary Table S1.pdf]

Supplementary Table S1. Distribution of anaphylaxis severity (WAO grades 1–5) by age and sex (male/female) in the study cohort. Values are patient counts.

	**Severity**	**1**	**2**	**3**	**4**	**5**
**Age (years)**	**Sex**					
0	male	0	1	1	0	0
	female	0	0	0	0	0
1	male	1	10	27	6	1
	female	0	6	7	0	0
2	male	3	9	20	1	0
	female	1	6	9	0	0
3	male	1	3	5	0	0
	female	2	2	5	0	0
4	male	0	5	3	0	0
	female	1	1	8	0	0
5	male	0	2	6	0	0
	female	0	3	1	0	0
6	male	0	0	2	0	0
	female	1	0	0	0	0
7	male	0	1	2	0	0
	female	0	0	0	0	0
8	male	1	1	3	0	0
	female	0	0	0	0	0
9	male	0	1	3	0	0
	female	0	0	1	0	0
10	male	1	1	1	0	0
	female	0	0	0	0	0
12	male	0	4	1	0	0
	female	0	0	0	0	0
14	male	0	0	1	0	0
	female	0	0	0	0	0
15	male	0	2	2	1	0
	female	0	0	2	0	0
16	male	0	0	3	0	0
	female	0	0	0	0	0
17	male	0	1	2	0	0
	female	0	1	2	0	0
18	male	0	0	0	0	0
	female	0	1	0	0	0
