# Supplementary material for: Nutritional Status Is Not a Predictor of Anaphylaxis Severity in a Pediatric Cohort: A Retrospective Analysis
Source: Nutrients. 2025 Sep 22;17(18):3023. doi: 10.3390/nu17183023 (PMC12472751; doi:10.3390/nu17183023)
Supplement: Supplementary file 1 [file nutrients-17-03023-s001.zip › Supplementary Table S2.pdf]

Supplementary Table S2. Association between BMI and anaphylaxis severity in primary and sensitivity analyses

Analysis	df	χ^2 statistic	p-value	Post hoc power
Primary (4 BMI categories × 5 WAO grades)	12	7.06	0.854	0.28
Sensitivity 1 (BMI 2 categories × 5 WAO grades)	4	3.57	0.469	0.20
Sensitivity 2 (BMI 2 categories × 2 severity levels)	1	0.68	0.411	0.09
